# Intracorporeal vs. extracorporeal open and closed knot tying techniques in laparoscopy: A randomized, controlled study

**DOI:** 10.1016/j.heliyon.2024.e25178

**Published:** 2024-01-26

**Authors:** Kathrin B. Labrosse, Claudia Marinho, Bernhard Fellmann-Fischer, Franziska Geissler, Andreas Schötzau, Viola Heinzelmann-Schwarz, Tibor A. Zwimpfer

**Affiliations:** a Department of Gynecological Oncology, University Hospital Basel, 4031 Basel, Switzerland; b Department of Biomedicine, University Basel and University Hospital Basel, 4031 Basel, Switzerland; c Medical Faculty, University Basel, 4056 Basel, Switzerland; d Peter MacCallum Cancer Centre, Melbourne, 3000, Victoria, Australia

**Keywords:** Laparoscopy, Education, Knot-tying techniques, Suturing techniques, Extracorporeal and intracorporeal knots, Box trainer, Validated exercise

## Abstract

**Objective:**

Tying knots during suturing is one of the most challenging tasks in laparoscopic surgery. Therefore, measures aimed at ensuring both the ease and speed of knot tying not only benefit the surgeon but can also reduce operating time significantly. This study compared extracorporeal and intracorporeal knot tying techniques using a Szabo pelvic trainer model from the Gynaecological Endoscopic Surgical Education and Assessment program.

**Design:**

The students tied intra- and extracorporeal knots using closed- and open-jaw knot pushers. Using an artificial tissue suturing pad in a certified Szabo pelvic trainer, students tied three knots using each technique according to block randomization. Task completion time, knot strength, knot-spread ability, and number of errors were recorded. The Wilcoxon test and mixed-effects models were used to analyze the results. After completing the exercises, participants answered a questionnaire concerning knot-tying techniques and their performance.

**Setting:**

University Hospital Basel, which provides tertiary-level clinical care.

**Participants:**

Fifty-seven medical students with no experience in laparoscopy voluntarily signed up for this study.

**Results:**

Open and closed extracorporeal knot tying was significantly faster (*p* < 0.001, p < 0.001, respectively), more precise (*p* = 0.007, *p* = 0.003), and associated with reduced knot-spread ability (*p* < 0.001, *p* < 0.001) compared to intracorporeal knot tying. Open- and closed-jaw knot pushers were shown to be equal in terms of speed (*p* = 0.563), knot-spread ability (*p* = 0.49), and precision (*p* = 0.831). The study participants rated open (30 %) and closed (49 %) extracorporeal knot tying as more intuitive than intracorporeal (21 %) knot tying. Improved concentration was significantly correlated with tighter knots (*p* = 0.011).

**Conclusions:**

Students achieved significantly better results using extracorporeal knot-tying techniques than intracorporeal ones, including greater speed, tighter knots, and optimized precision. These results suggest that beginners in the field of laparoscopy should be encouraged to practice extracorporeal knot-tying techniques.

## Highlights

• Extracorporeal knot tying resulted in greater speed and tighter knots than intracorporeal knot tying.
• Open- and closed-jaw knot pushers were shown to be equal with respect to speed and knot strength.
• Better concentration was correlated with significantly tighter knots.
• Non-experts find extracorporeal knot tying techniques more intuitive.

## 1 Introduction

Knot tying is crucial in surgical procedures, in which knots serve as mechanical ligatures between filaments [1]. While various laparoscopic knot types exist, the flat square knot remains the most popular and reliable, given that slip knots are generally significantly weaker [2]. However, in situations associated with considerable tension or in which the suture lacks sufficient friction to hold, the square-to-slip knot is particularly helpful [3,4]. Moreover, the square-to-slip knot can be easily manipulated in smaller spaces, such as the pelvis, and can be located carefully and precisely in the tissue [3].

Although knot tying for open surgical procedures can be easily taught, trained, and performed, it is considerably more challenging in laparoscopic surgery [5,6]. Knot tying during open surgery permits greater exposure of the surgical field, provides superior tactile sensation, and permits the use of all six degrees of freedom compared to laparoscopic surgery [1,4,5,7,8]. In laparoscopic surgery, however, the surgeon must confront various obstacles, including indirect visualization, loss of freedom of movement, fixed port positions, and limited working space [4]. Even experienced laparoscopic surgeons often consider knot tying to be difficult [1,5,[7], [8], [9], [10]].

Laparoscopic knot tying may be performed extracorporeally or intracorporeally, and both techniques have their respective advantages and disadvantages [11]. Intracorporeal knot tying is challenging, given its higher technical requirements and lower spatial availability than the extracorporeal approach [12]. Additionally, the surgeon experiences less tactile sensation in terms of the tension applied to the tissue and the knot [13]. By comparison, extracorporeally tied knots are technically easier to achieve [14]. However, in pulling long lengths of suture through the needle tract, the tissue may be affected, and attempts to push the knot into position may exert excessive tension on the tissue [15]. Moreover, the surgeon may pay less attention to the operative field during knot tying owing to the significantly smaller working field [16].

However, the ability to tie a knot with relative ease and speed not only benefits the surgeon but can also dramatically reduce operating time [17]. As healthcare costs increase, the optimization of surgical time has the potential to be pivotal in lowering these costs. It is thus crucial to investigate possible factors that may contribute to this [18]. In addition, surgical duration is correlated with postoperative infectious morbidity [19]. As such, it is imperative that the optimal laparoscopic knot-tying techniques be identified.

The existing literature discussing various laparoscopic suturing methods primarily compares intracorporeal knot tying with mechanical devices that can facilitate suturing procedures [20,21]. Most of these studies address different endpoints, such as knot strength, suture choice, and physician preference in terms of time, ease of use, and cost analysis. To the best of our knowledge, however, no study to date has examined time, knot strength, knot-spread ability, and subjective knot-tying preference in a certified pelvic trainer model [22].

The present study's aim was to compare laparoscopic extracorporeal and intracorporeal knot-tying techniques with respect to time, knot strength, and knot-spread ability when performing a square-to-slip knot. We hypothesized that because extracorporeal knot-tying techniques are less complex, non-experts would be able to perform them more easily, more swiftly, and with fewer mistakes.

## 2 Materials and methods

### 2.1 Power analysis

To estimate the required sample size of at least 49 subjects, we used a pragmatic approximation of the statistical distribution of task duration for the power analysis (two-sided paired t-tests with 90 % power and 0.05 significance level).

### 2.2 Study population

A total of 513 measurements per variable were obtained from 57 recruited students. We excluded 86 measurements across all variables due to participants making irreversible errors that included pulling the thread out of the stitch, irreversibly knotting the threads together or being unable to perform the knot after watching instructional videos.

The study participants were medical students (average age of 22 years) with no experience of laparoscopic surgery.

All study activities followed Institutional Review Board (IRB) guidelines for exempt studies, and all methods were performed according to the relevant guidelines and regulations. The Ethics Committee of Northwest and Central Switzerland (EKNZ) issued a formal IRB exemption certificate (Req-2021-01077) on September 21, 2021. The EKNZ is able to confirm that the research project (Req-2021-01077) is in compliance with the general ethical and scientific standards for research involving human subjects. Written informed consent to participate in the study was obtained from all participants. All personal data collected were anonymized.

### 2.3 Study design

**Fig. 1:**
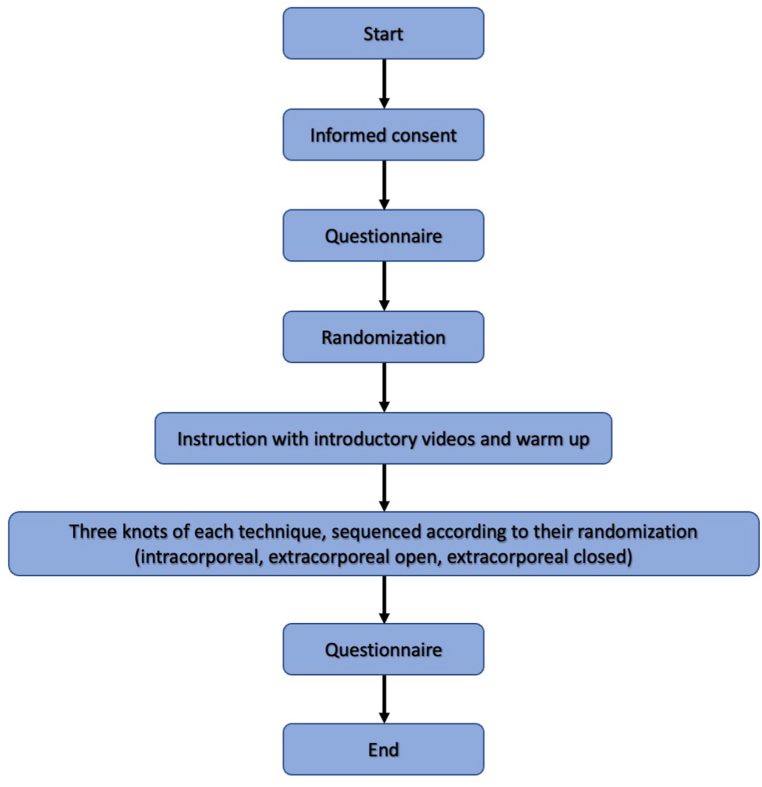
Flow diagram of the study design.

Fifty-seven participants were divided into randomized blocks of three knotting techniques with six different sequences of knot tying. Using closed and open jaw knot pusher, all participants performed intracorporeal and extracorporeal knots (Fig. 1). According to their randomization, they performed three knots of each technique. The primary outcome measure was the time to completion of the task during each participant's performance. After completing the task, the secondary endpoints 1) number of mistakes made, 2) knot strength, and 3) ability to spread the knot were measured. Participants were given questionnaires about their background and their experience of the exercises before and after completing the tasks.

### 2.4 Instructions

The participants were given a written outline describing the project prior to starting the exercises. The details of the procedures were explained in short sentences. The participants also watched an introductory video (Videos 1–3) before each task, which detailed the task's aim. Subjects were given 12 min to learn each technique and could pause and replay the video as often as needed within the given timeframe. Afterwards, they were shown the laparoscopic instruments and given a brief oral explanation of how to use them. If the participants had further questions, they were given the opportunity to ask them prior to the exercise's commencement.

### 2.5 Instrument set-up

The exercises were performed on a Gynaecological Endoscopic Surgical Education and Assessment (GESEA) certified pelvic trainer. A 24″ monitor, a Storz Hopkins II telescope (10 mm, 0°) with a Nova 300 light source, an Image 1 H3-Z Full HD camera (Karl Storz SE & Co., Tuttlingen) and a 300W xenon light source (Karl Storz SE & Co., Tuttlingen) were used. The instruments were inserted through two entry points corresponding to the lateral auxiliary trocar entry points.

### 2.6 Exercises

The technical knot-tying exercise was designed to assess different laparoscopic skills, including precision, speed, and dexterity. As the focus of the study was on knotting, stitching had been completed in advance by the principal investigator. To measure task completion time, areas were marked to define the starting position for the laparoscopic instruments. The task commenced in this position and was completed once the remains of the thread had been completely removed. A mobile phone with a start/stop feature was used to record the time that had elapsed at the end of every run and to measure the time required for each task's completion. Mistakes were manually counted and recorded. Four outcomes of interest were identified: (a) task time, (b) number of mistakes, (c) knot strength, and (d) knot-spread ability.

### 2.7 Intracorporeal knot

For this method, only two needle-holders (Geyi Medical 801.023) and laparoscopic scissors were needed. The selected knot, a square-to-slip knot [3,4], comprised three loops that were wound around the left needle holder with the help of the right needle holder. To produce the knot in accordance with the instructions in the introductory video, the first and third loops had to be wound clockwise and the second loop counterclockwise. The task was finished once the remaining threads had been truncated using laparoscopic scissors and completely removed from the pelvic trainer. If the loop directions were disregarded, the loop was wound around the right-hand instrument, or any thread remnants lost in the pelvic trainer, they were recorded as mistakes. In instances wherein the thread had been pulled all the way out of the stitch or the knot was cut open at the end, the run was terminated and documented as not valid. If an error occurred in the first run regarding the loops, the participant was alerted at the end of the task to pay better attention to the loop directions in the next run. We chose a slip square knot and this specific loop sequence in our study to make the knots more comparable, given that our priority was to evaluate the intra- and extracorporeal knot-tying techniques.

### 2.8 Extracorporeal knot, open jaw-type knot pusher

For this technique, an open jaw-type knot-pusher (Karl Storz 26596 D open jaw end) was provided in addition to the needle-holders and scissors. Initially, the needle had to be pulled out from the inside through the left-hand trocar. Subsequently, a square-to-slip knot was formed, comprising three loops that were individually pushed down with the open jaw-type knot-pusher. The first and third loops followed the same direction, whereas the second had to be pushed in the opposite direction. In addition, for the third loop, it was necessary to exchange the two holding threads. If the loop directions were not followed, the threads were not exchanged for the third loop, or if the thread's remnants were left in the pelvitrainer, they were recorded as mistakes. The performance of a second loop on top of a loop stuck in the trocar—thus forming a knot and stopping the run irreversibly—was deemed an invalid attempt.

### 2.9 Extracorporeal knot, closed jaw-type knot-pusher

This method required a closed jaw-type knot-pusher (Karl Storz 26596 D closed jaw end) and a Kocher clamp. First, the thread with the needle had to be pulled out of the left trocar. The needle was then removed, and the thread was threaded into the knot-pusher. This was followed by a square-to-slip knot consisting of three loops (previously described in knot-tying technique B). For the third loop, the threads had to be exchanged, which required opening the clamp to allow the old thread to be released and then threading the new thread and securing it again with the clamp. Mistakes were counted when participants disregarded the direction of the loop, did not exchange the threads, or threaded the wrong thread at the beginning.

### 2.10 Knot evaluation

Knot strength and knot-spread ability were used to evaluate knot quality. Knot strength was measured by elongation of the loop [23]. First, the length of one thread end was measured with a caliper gauge, and then the loop was pulled with a dynamometer at 15 N. Next, the thread was measured again, and the length difference was recorded in mm.

Knot-spread ability was determined by measuring the loop's dilatation after it was spread using curved scissors. A caliper gauge was used to record the dilatation in mm.

### 2.11 Questionnaires

Before and after the exercises, participants were asked to complete questionnaires. The pre-exercise survey covered general participant characteristics such as gender, age, video game playing habits and frequency, type of sport and instruments, and surgical and technical skill background. At the end of the exercises, the students completed a questionnaire to assess how they felt physically and mentally during the different knot-tying techniques. A numerical rating scale (NRS) of 0–10 was used for the questions, except for the demographic characteristics of the participants and the follow-up questions.

### 2.12 Statistical analysis

For categorical data, counts and frequencies, and for ordinal or metric variables, means with standard deviations, medians and interquartile ranges (IQRs) are presented. For the prediction of spreading ability, linear mixed effects models were used. Predictor variables were technique and run (first, second, or third run). For nested comparisons within runs, an interaction between technique and run was included in the models. The results are presented as mean differences. For total run time and delta knot strength, these variables were log transformed. Therefore, the results are presented as geometric mean ratios. For mistakes, values were dichotomized as 0, = /> 1 and a generalized linear mixed-effects model with logit link was applied. The results are presented as odds ratios. All estimators of the mixed models are presented with 95 % confidence intervals (CIs) and p-values. Associations between the questionnaire responses and the study variables were examined using the Kruskal–Wallis test or Spearman correlation, as appropriate. No adjustments were made for multiple comparisons. A p-value <0.05 was considered significant. Statistical analyses were performed using R version 4.1.3 statistical software.

## 3 Results

### 3.1 Time

**Fig. 2:**
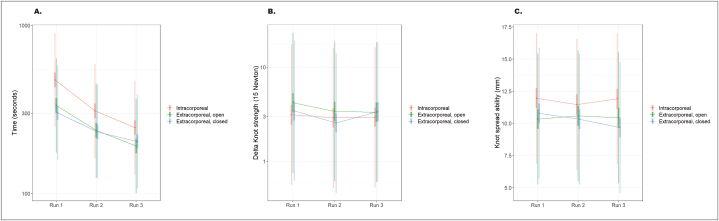
A comparison of the three knot tying techniques for all three runs for **A.** Time, **B.** Knot strength, and **C.** Knot-spread ability according to the geometric mean ratio.

Overall, the intracorporeal technique took significantly longer, with a mean time of 386.6 s, compared to the extracorporeal technique with the open jaw-type (279.5 s, *p* < 0.001) and the closed jaw-type (255.7 s, *p* < 0.001). No significant difference in time to task completion was observed between the extracorporeal techniques (*p* = 0.563), but the closed-type jaw was shown to take less time than the open-type jaw **(**Table 1**)**. This trend also emerged when the runs were compared individually (Fig. 2A). For all three techniques, participants improved their mean task completion time with every run and were significantly faster *(p <* 0.001) in completing the exercise during the second and third repetitions **(**Table 2**).**

**Table 1:** Comparison of different knot tying techniques by run time, knot strength and knot-spread ability.

			95 % Confidence Interval			
Parameter	Contrast	Ratio^a^ means		Lower	Upper	*p*-value^b^
Time (sec)	Intracorporeal: Extracorporeal, open	1.33 (386.6:279.5)		1.244	1.429	<0.001
	Intracorporeal: Extracorporeal, closed	1.361 (386.6:255.7)		1.27	1.458	<0.001
	Extracorporeal, open: Extracorporeal, closed	1.02 (279.5:255.7)		0.953	1.093	0.563
Knot strength (mm)	Intracorporeal: Extracorporeal, open	0.827 (3.80:4.92)		0.688	0.994	0.043
	Intracorporeal: Extracorporeal, closed	0.966 (3.80:4.96)		0.805	1.159	0.709
	Extracorporeal, open: Extracorporeal, closed	1.168 (4.92:4.96)		0.975	1.4	0.092
Knot-spread ability (mm)	Intracorporeal: Extracorporeal, open	1.328 (11.72:10.46)		0.782	1.875	<0.001
	Intracorporeal: Extracorporeal, closed	1.519 (11.77:10.26)		0.976	2.063	<0.001
	Extracorporeal, open: Extracorporeal, closed	0.191 (10.46:10.26)		−0.347	0.729	0.486
Mistakes	Intracorporeal: Extracorporeal, open	2.294 (0.36:0.28)		1.261	4.17	0.007
	Intracorporeal: Extracorporeal, closed	2.453 (0.36:0.22)		1.353	4.447	0.003
	Extracorporeal, open: Extracorporeal, closed	1.07 (0.28:0.22)		0.578	1.981	0.831

a For ‘Time’ and ‘Knot strength’, ratios correspond to geometric mean ratios. For ‘Knot-spread ability, ratios correspond to differences of means. For ‘Mistakes’, ratios correspond to odds ratios. The geometric mean ratio approximately corresponds to the median ratio.

b The p-values were calculated using a mixed-effects model. A p-value <0.05 was considered significant.

**Table 2:** Comparison of different knot tying techniques for each run by run time, knot strength, knot-spread ability, and mistakes made during the exercises.

				95 % Confidence interval		
Parameter	Run	Contrast	Ratio^a^ means	Lower	Upper	*p*-value^b^
Time (sec)	1	Intracorporeal: Extracorporeal, open	1.42 (541.0:373.9)	1.259	1.603	<0.001
		Intracorporeal: Extracorporeal, closed	1.569 (541.0:315.1)	1.391	1.769	<0.001
		Extracorporeal, open: Extracorporeal, closed	1.104 (373.9:315.1)	0.982	1.242	0.098
	2	Intracorporeal: Extracorporeal, open	1.307 (349.5:253.4)	1.16	1.473	<0.001
		Intracorporeal: Extracorporeal, closed	1.334 (349.5:242.4)	1.185	1.502	<0.001
		Extracorporeal, open: Extracorporeal, closed	1.021 (253.4:242.4)	0.907	1.149	0.735
	3	Intracorporeal: Extracorporeal, open	1.284 (269.2:211.1)	1.142	1.445	<0.001
		Intracorporeal: Extracorporeal, closed	1.211 (269.2:209.7)	1.077	1.361	0.001
		Extracorporeal, open: Extracorporeal, closed	0.943 (211.1:209.7)	0.838	1.06	0.325
Knot strength (mm)	1	Intracorporeal: Extracorporeal, open	0.746 (3.93:5.91)	0.541	1.029	0.074
		Intracorporeal: Extracorporeal, closed	0.909 (3.93:5.59)	0.661	1.252	0.56
		Extracorporeal, open: Extracorporeal, closed	1.219 (5.91:5.59)	0.891	1.668	0.215
	2	Intracorporeal: Extracorporeal, open	0.856 (3.69:4.73)	0.622	1.178	0.339
		Intracorporeal: Extracorporeal, closed	1.136 (3.69:3.65)	0.828	1.559	0.428
		Extracorporeal, open: Extracorporeal, closed	1.327 (4.73:3.65)	0.969	1.819	0.078
	3	Intracorporeal: Extracorporeal, open	0.883 (3.78:4.12)	0.645	1.21	0.439
		Intracorporeal: Extracorporeal, closed	0.870 (3.78:5.65)	0.636	1.191	0.384
		Extracorporeal, open: Extracorporeal, closed	0.985 (4.12:5.65)	0.720	1.348	0.926
Knot-spread ability (mm)	1	Intracorporeal: Extracorporeal, open	1.618 (12.01:10.31)	0.665	2.571	<0.001
		Intracorporeal: Extracorporeal, closed	1.166 (12.01:10.79)	0.217	2.114	0.016
		Extracorporeal, open: Extracorporeal, closed	−0.453 (10.31:10.79)	−1.381	0.476	0.34
	2	Intracorporeal: Extracorporeal, open	0.883 (11.42:10.60)	−0.065	1.831	0.068
		Intracorporeal: Extracorporeal, closed	1.143 (11.42:10.32)	0.204	2.081	0.017
		Extracorporeal, open: Extracorporeal, closed	0.26 (10.60:10.32)	−0.674	1.193	0.585
	3	Intracorporeal: Extracorporeal, open	1.467 (11.87:10.46)	0.534	2.401	0.002
		Intracorporeal: Extracorporeal, closed	2.236 (11.87:9.67)	1.307	3.166	<0.001
		Extracorporeal, open: Extracorporeal, closed	0.77 (10.46:9.67)	−0.16	1.698	0.105
Mistakes	1	Intracorporeal: Extracorporeal, open	2.338 (0.50:0.41)	0.938	5.83	0.068
		Intracorporeal: Extracorporeal, closed	1.74 (0.50:0.37)	0.721	4.199	0.218
		Extracorporeal, open: Extracorporeal, closed	0.744 (0.41:0.37)	0.299	1.853	0.526
	2	Intracorporeal: Extracorporeal, open	2.638 (0.37:0.20)	0.938	7.423	0.066
		Intracorporeal: Extracorporeal, closed	2.709 (0.37:0.21)	0.976	7.519	0.056
		Extracorporeal, open: Extracorporeal, closed	1.027 (0.20:0.21)	0.341	3.088	0.963
	3	Intracorporeal: Extracorporeal, open	1.836 (0.20:0.21)	0.617	5.462	0.275
		Intracorporeal: Extracorporeal, closed	4.411 (0.20:0.07)	1.212	16.05	0.024
		Extracorporeal, open: Extracorporeal, closed	2.403 (0.21:0.07)	0.628	9.2	0.201

a For ‘Time’ and ‘Knot strength’, ratios correspond to geometric mean ratios. For ‘Knot-spread ability’, ratios correspond to differences of means. For ‘Mistakes’, ratios correspond to odds ratios. The geometric mean ratio approximately corresponds to the median ratio.

b The p-values were calculated using a mixed-effects model. A p-value <0.05 was considered significant.

### 3.2 Knot strength

The intracorporeal technique led to a significantly higher knot strength, with a mean of 3.80 mm in contrast to the extracorporeal method with the open-jaw knot-pusher (mean 4.92 mm, *p* = 0.043) **(**Table 1**)**. The extracorporeal techniques showed no significant difference *(p =* 0.092) in knot strength, but a strong trend emerged indicating that the extracorporeal closed-type jaw (mean 4.96 mm) had a higher knot strength than the open-type jaw. These results did not suggest a significant difference between the intracorporeal technique and the extracorporeal closed-type jaw (*p* = 0.709). Overall, for the three techniques, an improvement in knot strength was evident from the first to the third run **(**Table 2**,** Fig. 2**B).**

### 3.3 Knot-spread ability

In terms of knot-spread ability, knots formed using the intracorporeal technique (mean of 11.77 mm) were significantly looser than those formed using the extracorporeal open-type jaw (10.46 mm, *p* < 0.001) and the closed-type jaw (10.26 mm, *p* < 0.001). However, no significant difference emerged between the two extracorporeal techniques (*p* = 0.49). These results were also reflected in the comparisons of individual runs (Fig. 2C). One exception was during the second run, when the intracorporeal method and the extracorporeal open jaw-type showed no significant difference (*p* = 0.068); however, there remained a strong trend indicating that the extracorporeal open jaw-type had lower knot-spread ability than the intracorporeal technique and, hence, a tighter knot. Overall, all three techniques showed a decrease in knot-spread ability with every run. The intracorporeal technique had a mean of 12.01 mm in the first run, 11.42 mm in the second, and 11.87 mm in the third run. The extracorporeal open jaw-type had 10.31 mm in the first run, 10.60 mm in the second, and 10.46 mm in the third run. The extracorporeal closed-type jaw had a knot-spread ability of 10.79 mm in the first run, 10.32 mm in the second run, and 9.67 mm in the third run **(**Table 1**)**.

### 3.4 Precision

**Fig. 3:**
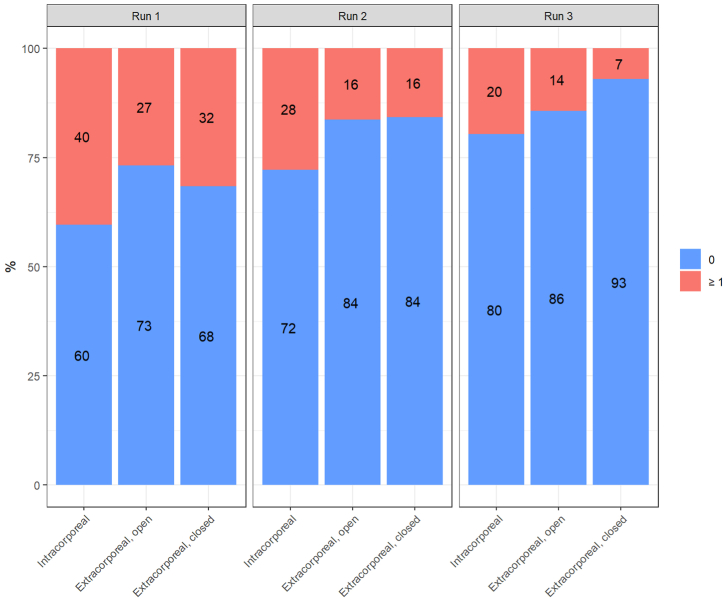
The mistakes made for each knot-tying technique over all three runs.

The intracorporeal technique led to significantly more faulty trials, with an odds ratio of 2.453 compared to the extracorporeal closed jaw-type *(p* = 0.003), and an odds ratio of 2.294 compared to the open jaw-type (*p* = 0.007) (Table 2). By contrast, mistakes made when performing the two extracorporeal techniques did not differ significantly from one another *(p* = 0.831). When the runs were compared individually, most of the faulty trials in the first run occurred while performing the intracorporeal technique (40 % of the trials), followed by the extracorporeal closed-type jaw (32 %), and the extracorporeal open-type jaw (27 %) (Fig. 3). The occurrence of mistakes decreased with every run. The intracorporeal technique resulted in more faulty trials across all attempts, decreasing from 40 % in the first run to 28 % in the second and 20 % in the final run. Both extracorporeal techniques had the same number of faulty trials in the second run (16 %). In the third and final run, the closed jaw-type had the fewest faulty trials (7 %), compared to the open jaw-type (14 %).

### 3.5 Pre-exercise questionnaire results

The parameters gender, age, current semester, and leisure activities that require physical skills, such as playing ball sports, a musical instrument, or video games showed no significant influence on the four outcomes of interest. However, knot strength showed a trend in differences between genders *(p* = 0.077), with a higher knot strength in those made by males. No significant difference was evident in the results concerning whether the participants intended to work in a surgical or medical field in the future **(**Table 3**)**.

**Table 3:** Correlations between the parameters from the questionnaire and the outcomes of time run, knot strength, and knot-spread ability.

Parameter	Outcome	*p*-value^a^
Gender	Time	0.69
	Knot strength	0.077
	Knot-spread ability	0.620
Age	Time	0.83
	Knot strength	0.415
	Knot-spread ability	0.637
Semester	Time	0.221
	Knot strength	0.608
	Knot-spread ability	0.163
Specialty	Time	0.499
	Knot strength	0.575
	Knot-spread ability	0.108
Video games	Time	0.651
	Knot strength	0.319
	Knot-spread ability	0.839
Ball sports	Time	0.313
	Knot strength	0.107
	Knot-spread ability	0.212
Musical instrument	Time	0.215
	Knot strength	0.911
	Knot-spread ability	0.471
Average concentration	Time	0.408
	Knot strength	0.063
	Knot-spread ability	0.011
Intuitiveness	Time	0.117
	Knot strength	0.628
	Knot-spread ability	0.477

a The p-values were calculated using Kruskal-Wallis test or Spearman correlation as appropriate. A p-value <0.05 was considered significant.

### 3.6 Post-exercise questionnaire results

Participants rated the intracorporeal knot-tying technique as the most challenging, followed by the extracorporeal method with the open-jaw knot-pusher and the extracorporeal technique with the closed-jaw knot-pusher. Furthermore, the participants found it slightly easier to familiarize themselves with the extracorporeal knot-tying technique using the closed-jaw knot-pusher than the other two techniques. Their ability to maintain concentration was identical across all three techniques. In addition, knot-spread ability was significantly decreased with a higher level of participant concentration *(p* = 0.011).

**Fig. 4:**
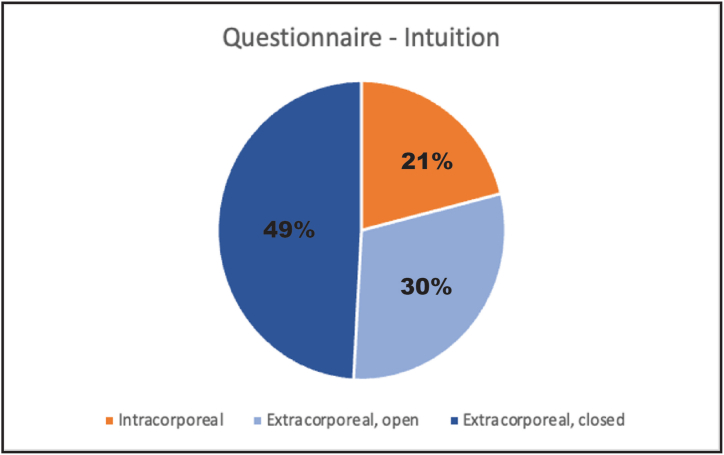
Comparison of the three knot-tying techniques.

When asked which technique they found to be most intuitive, 49 % of participants chose the extracorporeal knot-tying technique with the closed-jaw knot-pusher, followed by the extracorporeal knot-tying technique with the open-jaw knot-pusher (30 %) (Fig. 4). The participants based their decisions on various criteria, including ease of handling, complexity, and rapid customization.

## 4 Discussion

The study's findings suggest that extracorporeal knot-tying techniques carry key benefits relative to intracorporeal knot tying. Such benefits include greater speed, tighter knots, and optimized precision with fewer mistakes. No difference was observed in terms of the sequence in which the techniques were implemented, and students improved their learning curve for all tasks. This study included students who had no prior experience with laparoscopy to better evaluate the optimal knot-tying techniques for beginners in the field of minimally invasive surgery. The study was conducted on a certified Szabo pelvic trainer, which is the validated trainer box used in the GESEA programme, with an artificial tissue suturing pad that mimics human flesh to ensure that the exercises achieved criterion validity and content validity [24]. Additionally, the exercises reached face validity when rated by experts as valuable in terms of realistic laparoscopic training [25].

The extracorporeal knot-tying techniques resulted in significantly faster performance, with greater precision than the intracorporeal knot-tying technique. Moreover, the knots were tighter. The two different extracorporeal knot-tying techniques showed no difference with respect to speed, precision, or knot-spread ability. These results support the hypothesis that extracorporeal knot tying is simpler, safer, more reproducible, and—above all—does not require any special skill or great manual dexterity, given that it is performed externally [26]. However, despite these advantages, this technique cannot be used for all tissue types, as it exerts excessive tension on the tissue [15].

Regarding the primary endpoint ‘speed,’ participants improved their performance in the second and third runs, regardless of the technique used, which suggests a steep learning curve [27]. Knot-spread ability using the intracorporeal technique and the extracorporeal open jaw-type was nearly constant across all runs, whereas the extracorporeal technique with the closed jaw-type showed increasingly tighter knots throughout the runs. Knot strength showed no significant improvement or deterioration throughout the runs in accordance with the different knot-tying techniques. Despite the amelioration of the performance throughout the runs, both extracorporeal knot-tying techniques remained swifter and were associated with tighter knots.

The laparoscopy field continues to develop rapidly, and new methods using improved equipment are constantly being introduced [28]. While the learning curve for minimally invasive suturing has a shorter task-time curve when using robotic assistance compared to the laparoscopic learning curve, laparoscopic outcomes show good end results, with rapid outcome improvement [29]. Nevertheless, young assistant doctors do not typically have many opportunities to participate in robotic-assisted laparoscopies. It is thus important to provide an easy, fast, and reliable conventional laparoscopic knot-tying technique for beginners in the field. Simulation-based training has been shown to be successful and transferable to the operating theater [30,31]. Given that the traditional apprentice–tutor model is no longer sufficient for developing all the skills required for endoscopic surgery, it is clearly necessary that an education model, such as the European “Gynaecological Endoscopic Surgical Education and Assessment” (GESEA) or the American “Fundamentals of Laparoscopic Surgery” (FLS) program, be introduced as a core pillar of the surgical curriculum [32,33]. In particular, the intracorporeal knot-tying technique benefits from these simulation training sessions, since the actual technique used to tie an extracorporeal knot is regularly taught and used in open surgery [34]. For this reason, senior surgeons may even be in a position to entrust junior residents with extracorporeal knots in the operating room at an earlier stage.

The questionnaire responses confirmed our hypothesis that non-experts who have no previous experience in laparoscopic surgery would find the intracorporeal knot-tying technique more challenging and the extracorporeal techniques more intuitive (Fig. 4). Moreover, better concentration was correlated with significantly tighter knots *(p* = 0.011). This supports the idea that operating time may be reduced by choosing a fast and safe knot-tying technique. No difference was observed with respect to gender when the participants completed the tasks, although we did observe a strong trend where knots executed by men had superior strength *(p* = 0.077) [35]. Further findings from the questionnaire were in accordance with findings reported in the literature, suggesting that the superiority of extracorporeal knot-tying techniques is independent of gender, age, or the extent to which a student has played video games and/or musical instruments or the types of sports that they play [36].

Our study had several limitations. One was the *ex-vivo* study set-up, as there was no comparison with improvement in the operating room. To overcome this limitation, we used the GESEA-certified pelvic trainer, a model that has been validated for studies in the field of laparoscopy [24]. Another limitation concerns the lack of experts with experience in laparoscopy. However, we decided to include students exclusively for the following reasons: (1) the definition of ‘expert’ in the field of minimally invasive surgery is ambiguous, and the more experience a surgeon has, the greater their mastery over their preferred knot-tying technique is likely to be. This may have resulted in a bias in the data; (2) recruitment of the necessary sample size of experts would have been difficult and, with only a small number of experts, our study's potential would have been remarkably decreased; and (3) one of the study's strengths was its extensive data set, which included more than 500 measurements from a highly homogenous cohort. Another general limitation concerning the evaluation of training exercises is the learning effect. This was shown with improvements in run times for all three knot-tying techniques with every run. However, to prevent the bias of the learning curve on the outcome of the comparison of the knot techniques, we included students without experience in laparoscopy, and thus the findings might show that extracorporeal knot techniques showed a consistently improved outcome compared to intracorporeal knot techniques, regardless of the learning curve. Additionally, we implemented block randomization to prevent selection bias. Finally, different results may have been achieved had we used other types of needle-holders or knot-pushers, but the materials used in our study are considered to be standard laparoscopic instruments.

## 5 Conclusion

In summary, this study suggests that beginners in the field of laparoscopy should be encouraged to use extracorporeal knot-tying techniques, which are associated with faster and tighter knots than the intracorporeal knotting technique. Future investigations will entail a competency assessment that correlates participants’ individual performances in the operating theater.

## Ethics approval and consent to participate

All study activities were conducted in accordance with Institutional Review Board (IRB) guidelines for exempt studies. All methods were implemented in accordance with the relevant guidelines and regulations. A formal IRB certification of exemption (Req-2021-01077) was provided by the ethics committee of Northwest and Central Switzerland (EKNZ) on September 21, 2021. The EKNZ can confirm that the research project (Req-2021-01077) fulfilled the general ethical and scientific standards for research with human subjects. All participants gave their written informed consent to participate in the study. The anonymization of personal data was guaranteed.

## Funding

This work was supported by the Swiss National Foundation [P500PM_20726/1, 2021]; Bangerter-Rhyner Stiftung [0297, 2021]; and Freie Gesellschaft Basel [2022].

## Availability of data and material

The datasets analyzed for this study are available at https://datadryad.org/stash/share/s0FvMwxMdAWzgU5ir6WA1mOq_JOBvtOJ6eHqEnxtaXM.

## CRediT authorship contribution statement

**Kathrin B. Labrosse:** Writing – review & editing, Writing – original draft, Investigation, Formal analysis, Data curation, Conceptualization. **Claudia Marinho:** Writing – review & editing, Writing – original draft, Project administration, Methodology, Investigation, Data curation. **Bernhard Fellmann-Fischer:** Writing – review & editing, Writing – original draft, Visualization, Resources, Methodology, Investigation. **Franziska Geissler:** Writing – review & editing, Writing – original draft, Methodology, Investigation. **Andreas Schötzau:** Writing – review & editing, Writing – original draft, Visualization, Methodology, Investigation. **Viola Heinzelmann-Schwarz:** Writing – review & editing, Writing – original draft, Resources, Project administration, Funding acquisition. **Tibor A. Zwimpfer:** Writing – review & editing, Writing – original draft, Visualization, Supervision, Resources, Project administration, Methodology, Investigation, Funding acquisition, Formal analysis, Conceptualization.

## Declaration of competing interest

The authors declare that they have no known competing financial interests or personal relationships that could have appeared to influence the work reported in this paper.
