# Supplementary material for: Intracorporeal vs. extracorporeal open and closed knot tying techniques in laparoscopy: A randomized, controlled study
Source: Heliyon. 2024 Jan 26;10(3):e25178. doi: 10.1016/j.heliyon.2024.e25178 (PMC10844269; doi:10.1016/j.heliyon.2024.e25178)
Supplement: Multimedia component 1 [file mmc1.docx]

**Video 1.** Introductory video of the intracorporeal knot exercise <https://vimeo.com/783797757>
