# Supplementary material for: Intracorporeal vs. extracorporeal open and closed knot tying techniques in laparoscopy: A randomized, controlled study
Source: Heliyon. 2024 Jan 26;10(3):e25178. doi: 10.1016/j.heliyon.2024.e25178 (PMC10844269; doi:10.1016/j.heliyon.2024.e25178)
Supplement: Multimedia component 4 [file mmc4.docx]

**SUPPLEMENTARY TABLE 1.** Overall descriptive statistics of the measured parameters for all three laparoscopic knot techniques for every run.

| **Parameter** | **Technique** | **Run** | **median** | **mean** | **SD^a^** | **IQR^b^** |
| --- | --- | --- | --- | --- | --- | --- |
| Total time (sec) | Intracorporeal | 1 | 442.0 | 541.000 | 314.834 | 400.75 |
|  |  | 2 | 267.0 | 349.509 | 206.881 | 185.00 |
|  |  | 3 | 235.0 | 269.228 | 139.654 | 109.00 |
|  | Extracorporeal, open | 1 | 302.5 | 373.911 | 207.298 | 220.50 |
|  |  | 2 | 231.0 | 253.382 | 101.106 | 101.00 |
|  |  | 3 | 186.0 | 211.143 | 109.086 | 105.50 |
|  | Extracorporeal, closed | 1 | 298.0 | 315.053 | 94.471 | 114.00 |
|  |  | 2 | 227.0 | 242.421 | 77.594 | 73.00 |
|  |  | 3 | 200.0 | 209.737 | 49.101 | 62.00 |
| Knot strength (mm) | Intracorporeal | 1 | 3.30 | 3.925 | 3.214 | 2.425 |
|  |  | 2 | 2.70 | 3.693 | 3.396 | 2.150 |
|  |  | 3 | 2.70 | 3.782 | 3.254 | 3.350 |
|  | Extracorporeal, open | 1 | 4.65 | 5.913 | 5.109 | 6.250 |
|  |  | 2 | 3.00 | 4.733 | 3.933 | 4.600 |
|  |  | 3 | 3.60 | 4.115 | 2.689 | 3.125 |
|  | Extracorporeal, closed | 1 | 4.80 | 5.59 | 4.727 | 6.100 |
|  |  | 2 | 2.40 | 3.654 | 3.120 | 4.300 |
|  |  | 3 | 3.70 | 5.646 | 5.631 | 6.300 |
| Knot-spread ability  (mm) | Intracorporeal | 1 | 11.80 | 12.006 | 3.267 | 3.700 |
|  |  | 2 | 10.60 | 11.419 | 3.142 | 4.050 |
|  |  | 3 | 11.60 | 11.873 | 3.148 | 3.500 |
|  | Extracorporeal, open | 1 | 10.25 | 10.313 | 2.623 | 2.700 |
|  |  | 2 | 10.80 | 10.602 | 2.637 | 4.150 |
|  |  | 3 | 10.25 | 10.457 | 2.333 | 2.825 |
|  | Extracorporeal, closed | 1 | 10.80 | 10.791 | 3.282 | 4.000 |
|  |  | 2 | 10.30 | 10.323 | 2.555 | 2.600 |
|  |  | 3 | 9.60 | 9.672 | 2.600 | 3.100 |
| mistakes | Intracorporeal | 1 | 0 | 0.500 | 0.672 | 1 |
|  |  | 2 | 0 | 0.370 | 0.653 | 1 |
|  |  | 3 | 0 | 0.196 | 0.401 | 0 |
|  | Extracorporeal, open | 1 | 0 | 0.411 | 0.804 | 1 |
|  |  | 2 | 0 | 0.200 | 0.487 | 0 |
|  |  | 3 | 0 | 0.214 | 0.624 | 0 |
|  | Extracorporeal, closed | 1 | 0 | 0.368 | 0.587 | 1 |
|  |  | 2 | 0 | 0.211 | 0.559 | 0 |
|  |  | 3 | 0 | 0.070 | 0.258 | 0 |

*^a^sd, standard deviation*

*^b^IQR, interquartile range*
